# Experiential ownership and body ownership are different phenomena

**DOI:** 10.1038/s41598-021-90014-y

**Published:** 2021-05-19

**Authors:** Caleb Liang, Wen-Hsiang Lin, Tai-Yuan Chang, Chi-Hong Chen, Chen-Wei Wu, Wen-Yeo Chen, Hsu-Chia Huang, Yen-Tung Lee

**Affiliations:** 1 Department of Philosophy, National Taiwan University, Taipei, Taiwan; 2 Graduate Institute of Brain and Mind Sciences, National Taiwan University, Taipei, Taiwan; 3 Graduate Institute of Digital Learning and Education, National Taiwan University of Science and Technology, Taipei, Taiwan; 4 Department of Law, National Taiwan University, Taipei, Taiwan; 5 Department of Economics, National Taiwan University, Taipei, Taiwan; 6 Department of Philosophy, Western University, London, Canada; 7 Rotman Institute of Philosophy, Western University, London, Canada

**Keywords:** Psychology, Human behaviour

## Abstract

Body ownership concerns what it is like to feel a body part or a full body as mine, and has become a prominent area of study. We propose that there is a closely related type of bodily self-consciousness largely neglected by researchers—*experiential ownership*. It refers to the sense that *I* am the one who is having a conscious experience. Are body ownership and experiential ownership actually the same phenomenon or are they genuinely different? In our experiments, the participant watched a rubber hand or someone else’s body from the first-person perspective and was touched either synchronously or asynchronously. The main findings: (1) The sense of body ownership was hindered in the asynchronous conditions of both the body-part and the full-body experiments. However, a strong sense of experiential ownership was observed in those conditions. (2) We found the opposite when the participants’ responses were measured *after* tactile stimulations had ceased for 5 s. In the synchronous conditions of another set of body-part and full-body experiments, only experiential ownership was blocked but not body ownership. These results demonstrate for the first time the double dissociation between body ownership and experiential ownership. Experiential ownership is indeed a distinct type of bodily self-consciousness.

## Introduction

When I ride a bicycle, my hands gently hold the handlebar and feel the texture of its rubber surface; my legs take turns pushing the pedals and sense the resistance from them; as I speed up, I feel the wind on my face and feel that my body is moving forward a bit faster. Two kinds of bodily self-consciousness are involved in this simple example. First, I experience the hands on the handlebar as *my* hands, the legs pushing the pedals as *my* legs, and the whole body that is moving forward with the bike as *my* body. This is the sense of *body ownership*, which concerns what it is like to feel a body part or a whole body as mine. Second, I have an implicit sense that I am the unique *subject* of the experiences described above. That is, I feel that it is *me* who is experiencing the texture of the handlebar, it is *me* who is sensing the resistance from the pedals, and it is *me* who is feeling the wind on the face, etc. This is the sense of *experiential ownership*, the sense that I am the one who is having these experiences. In this study, we intend to investigate the relation between these two kinds of bodily self-consciousness. Are they one and the same? Or are they genuinely different phenomena?

Body ownership has been widely studied by using the paradigms of the rubber hand illusion (RHI)^1,2^ and full-body illusions (FBI)^3–5^. Many aspects of body ownership have been investigated, not only in real but also in virtual environments^6–8^. Various factors could affect whether an illusory sense of body ownership would be induced or abolished, including temporal, spatial anatomical constraints, and first- versus third-person perspectives, etc.^4,9–12^. Studies have indicated that under suitable manipulations body ownership could exhibit a high degree of flexibility^3,12–16^. Possible neural mechanisms of body ownership have also been suggested^11,17–21^.

By contrast, most discussions on experiential ownership up until now come from philosophers. For example, experiential ownership is associated with the idea of “self-as-subject” in the philosophy of language^22–24^. It is also related to the notion of “phenomenal self” in the traditional and interdisciplinary philosophy of mind^25–30^. When introducing this notion, Metzinger poses the following questions: “Why is there always someone having the experience? Who is the feeler of your feelings and dreamer of your dreams? Who is the agent doing the doing, and what is the entity thinking your thoughts? Why is your conscious reality your conscious reality?”^26^ These questions highlight the importance and ubiquity of the *who*-aspect embedded in conscious experience. We may say that one has the sense of experiential ownership when one has the sense of this *who*-aspect in an experience.

In this study, we intend to investigate the relation between body ownership and experiential ownership in the domain of bodily experience. The issue to be addressed is: Are body ownership and experiential ownership different types of bodily self-consciousness or are they just two aspects of the same phenomenon? As the example above indicates, body ownership concerns whether a body part or a whole body is experienced as mine, i.e., it is about *what* belongs to me. Experiential ownership, on the other hand, is about *who* is undergoing the relevant experiences. At first glance they seem to be different. But things are more complicated than that. In contrast to the large amount of literature on body ownership, experiential ownership is largely neglected by empirical researchers (for exceptions and discussions, see^31–34^). From an interdisciplinary standpoint, it is not enough to just learn about whether body ownership and experiential ownership are conceptually distinguishable. We intend to conduct experiments to see whether they are *empirically dissociable*.

Why is this issue important? On the one hand, if experiential ownership and body ownership turn out to be two aspects of the same phenomenon, then there is a need to explain how these two aspects are related to each other. Such an explanation would deepen our current understanding of body ownership. On the other hand, if experiential ownership is genuinely different from body ownership, this would mean that we identify an important phenomenon that has been largely overlooked by empirical researchers and thus open up a new sub-field in the study of bodily self-consciousness. Furthermore, experiential ownership is not only embedded in ordinary conscious experiences but is also relevant to neuropathology. Certain perplexing syndromes such as somatoparaphrenia^35–37^ are likely to involve defective senses of experiential ownership and could not be easily explained only in terms of impaired senses of body ownership. Consider the following two cases of somatoparaphrenia.

The first was a patient (F.B.) reported by Bottini et al.^35^: due to right hemisphere lesions, F.B. denied ownership of her left hand and insisted that it was her niece’s hand. She also had tactile extinction in her left hand and unilateral neglect in the left visual field. When she was blindfolded, the examiner gave her verbal hints and then touched the dorsal surface of her left hand. When told that her left hand would be touched, F.B. never reported feeling the touches. Surprisingly, when told that the examiner was going to touch her *niece’s* hand, she reported feeling the touches (70%, 70%, 100%, and 80% of the trials in four sessions). Bottini et al. said that “her tactile imperceptions dramatically recovered.” According to one interpretation, F.B.’s case involves misrepresentation of experiential ownership in addition to impaired body ownership^34^.

Moro et al.^36^ reported another two patients who also denied ownership of their left hand, lost their left visual field and could not feel tactile stimulations in their left hand. Surprisingly, when their left hand was moved across the corporeal midline to the right so that they could see it, they became capable of tactile sensations. The most interesting thing is that “stimuli were detected in all trials even though the hand was still felt as belonging to another person”^36^. Despite considering themselves as having tactile sensations, the patients still denied ownership of their left hand. This seems to suggest that it is possible to experience experiential ownership without body ownership.

These pathological cases are clearly relevant to whether experiential ownership and body ownership are different phenomena. However, cases like these are rare, and interpretations of them tend to be controversial. They can provide a research hint but will not settle the issue. Therefore, it is important to investigate this issue by experimenting on healthy participants. We conducted a series of experiments by adopting the RHI and the FBI paradigms^3,4^. The participants saw a fake hand or someone else’s body from the first-person perspective (1PP) and were touched either synchronously or asynchronously. With our new questionnaires, we compared the subjects’ sense of experiential ownership with their sense of body ownership in two different directions. (1) We tested the hypothesis that it is possible for the participants to have a clear sense of experiential ownership when their sense of body ownership was hindered. (2) In another set of experiments, the participants’ responses were measured after the tactile stimulations had stopped for a few seconds. Following previous studies, we assumed that an illusory sense of body ownership could be maintained for a few seconds without tactile stimulation^2,38,39^. We tested the hypothesis that, in the synchronous conditions, a positive sense of body ownership could still be detected even when the sense of experiential ownership had vanished. If both hypotheses are confirmed, they would show that body ownership and experiential ownership are doubly dissociable, and that experiential ownership is indeed a distinct phenomenon open to empirical research.

## Results

In Experiments 1 and 2 (Table 1), we tested the hypothesis that it is possible for a participant to experience experiential ownership without body ownership. In Experiments 3 and 4, we tested the opposite hypothesis that it is possible for a subject to experience body ownership without experiential ownership. Data were gathered by questionnaires and Skin conductance response (SCR) measurements. For the questionnaires (Table 2), seven questions were asked in each experiment: Q1 was about body ownership, supported by the touch-referral question, Q2. Q3 and Q5 were the main questions of experiential ownership. Q4 and Q6 were questions to rule out potential confounding factors from Q3 and Q5, respectively. Q7 was the control question. SCR was generally considered as reliable evidence of body ownership experience. Following the previous literature, we adopted the SCR method in Experiments 1~  4, which was measured 5 s after a knife threat was presented to the participants. In Experiments 5 and 6, we designed different procedures to measure experiential ownership with the SCR method. Here we present the main experimental results. For more details, see the Supplementary Information.

**Table 1:** Overview of Experiments.

Experiments	Description	Measurement	Participants (n)	Statistics
Exp. 1	Body part Sync. versus Async	Questionnaire & SCR	n = 34 (♂16) M = 21.7 ± 2.3	Wilcoxon signed-rank tests (Sync. versus Async.)
Exp. 2	Full Body Sync. versus Async	Questionnaire & SCR	n = 30 (♂15) M = 21.6 ± 2.6	Wilcoxon signed-rank tests (Sync. versus Async.)
Exp. 3	Body part Delayed measurement Sync. versus Async	Questionnaire & SCR	n = 30 (♂18) M = 23.6 ± 5.1	Wilcoxon signed-rank tests (Sync. versus Async.)
Exp. 4	Full body Delayed measurement Sync. versus Async	Questionnaire & SCR	n = 30 (♂15) M = 22.3 ± 2.9	Wilcoxon signed-rank tests (Sync. versus Async.)
Exp. 5	Synchronous brushing Touch versus No-touch	SCR	n = 18 (♂9) M = 21.1 ± 1.8	Wilcoxon signed-rank tests (Touch versus No-touch)
Exp. 6	Asynchronous brushing Touch versus No-touch	SCR	n = 16 (♂7) M = 21.8 ± 2.4	Wilcoxon signed-rank tests (Touch versus No-touch)

**Table 2:** Questionnaires.

Experiment 1 (body-part)/Experiment 2 (full-body)	
Q1	It felt as if I was looking at my hand/body
Q2	The touch that I felt was caused by the paintbrush/wood stick in front of me
Q3	During the experiment it was me who felt touched
Q4	During the experiment it was me who felt pain/tickled
Q5	I felt that I was being touched during the experiment
Q6	I felt that I was being hit/tickled during the experiment
Q7	It felt as if the hand/body in front of me gradually became a flower
**Experiment 3 (body-part)**/**Experiment 4 (full-body)**	
Q1	Right now, it feels as if I am looking at my hand/body
Q2	The touch that I felt was caused by the paintbrush/stick in front of me
Q3	Right now, it seems that it is me who is feeling touched
Q4	It seems that it was me who felt touched a moment ago
Q5	I am feeling touched right now
Q6	I felt that I was touched a moment ago
Q7	It felt as if the hand/body in front of me gradually became a flower

### Experiment 1: Body part

**Figure 1:**
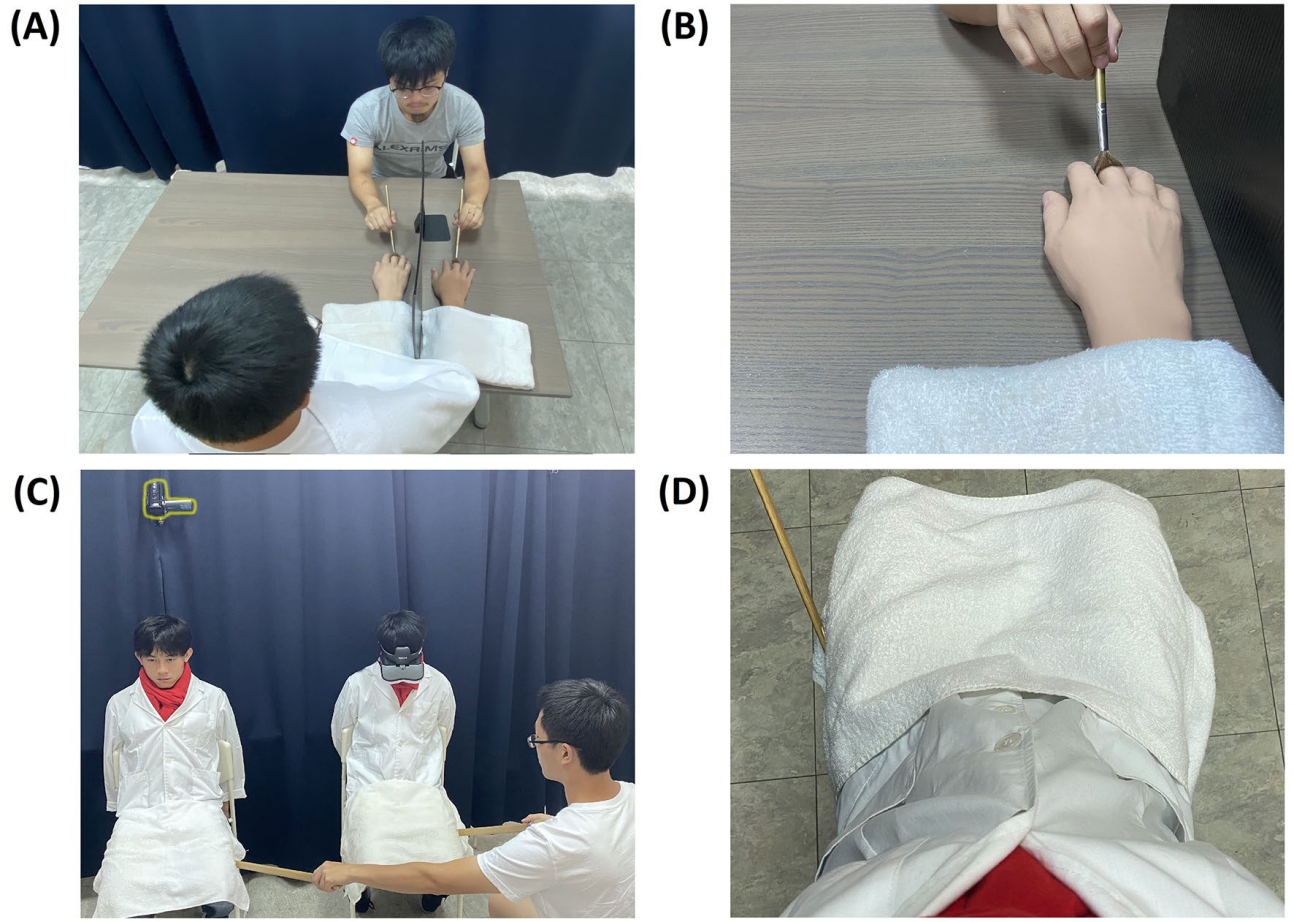
The Experimental Settings. (**A**) The setting of Experiments 1, 3, 5, and 6. The participant placed his/her right hand on a desk and the hand was blocked from view. Instead, he/she saw a rubber hand in front. During the experiment, the participant’s real hand was brushed synchronously or asynchronously with respect to the rubber hand. (**B**) Image seen from the participants’ 1PP in Experiments 1, 3, 5, and 6. (**C**) The setting of Experiments 2 and 4. The participant wore a lab coat, sat on a chair and put on an HMD connected to a real-time camera filming an experimenter’s body. What the participant saw from the adopted 1PP was actually the experimenter’s body. During the experiment, a second experimenter tapped both the first experimenter’s and the participant’s left leg synchronously or asynchronously. (**D**) Image seen from the participants’ adopted 1PP in Experiments 2 and 4.

**Figure 2:**
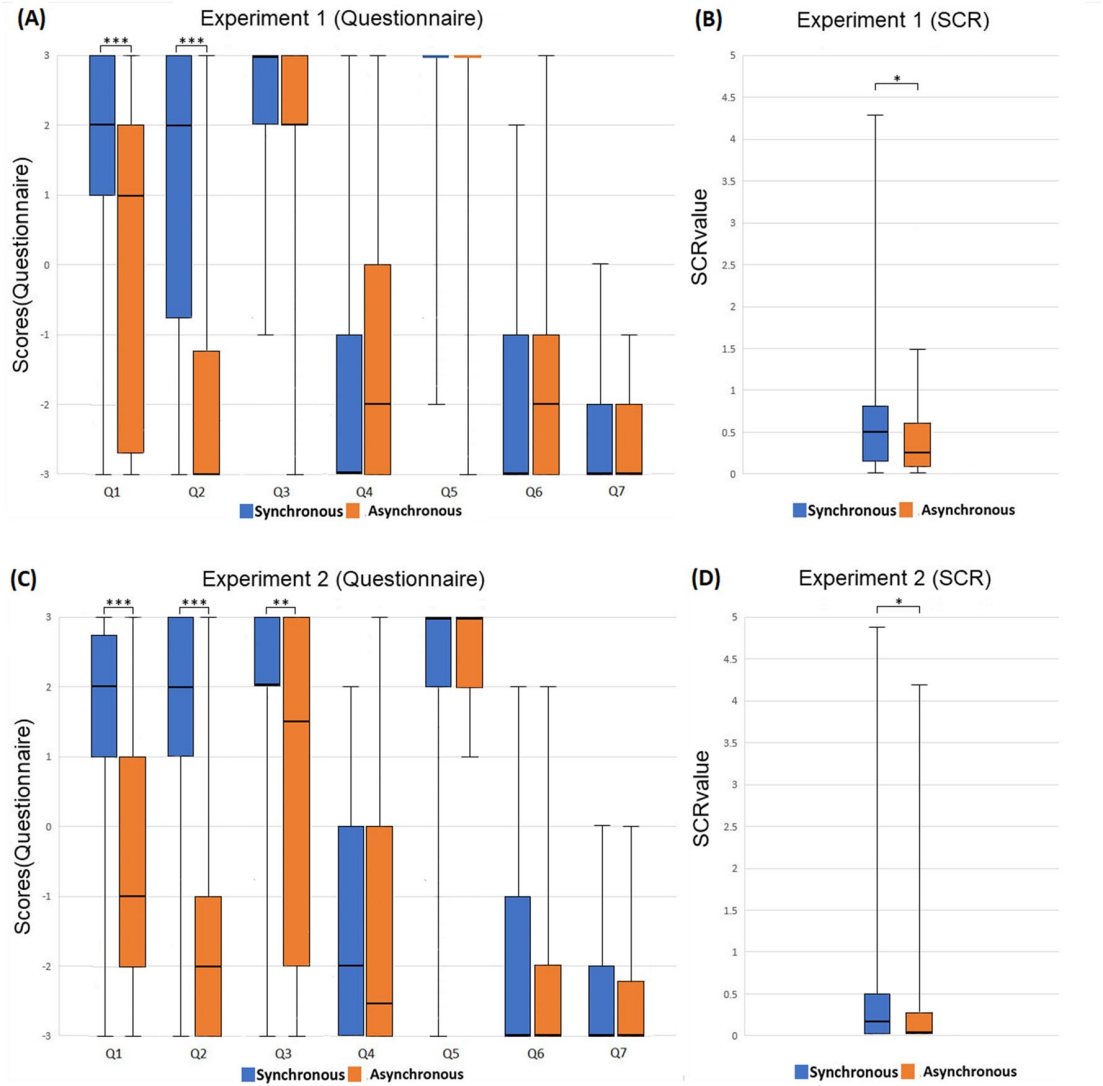
Results of Experiments 1 and 2. (**A**) The box chart of each question of Experiment 1. (**B**) The box chart of SCR values of Experiment 1. (**C**) The box chart of each question of Experiment 2. (**D**) The box chart of SCR values of Experiment 2. The error bars represent the maximum/minimum value. SCR value = (∆SCR in SP)/(SCR value range in RP). Significance levels: * p < 0.05; ** p < 0.01; *** p < 0.001.

In Experiment 1, we performed the standard RHI experiment. The participant watched the fake hand from the 1PP and was brushed either synchronously or asynchronously (Fig. 1A,B). The box charts of each question and SCR values are shown in Fig. 2A,B. By two-tailed Wilcoxon signed-rank test, Q1 (Z = 3.353, *p* < 0.001, effect r = 0.407), Q2 (Z = 4.117, *p* < 0.001, effect r = 0.499) and the SCR results (Z = 2.368, *p* = 0.017, effect r = 0.287) were significant between the synchronous/asynchronous manipulations, confirming the consensus on body ownership in the literature. Regarding experiential ownership, both questions showed very high medians, not only in the synchronous condition (sync. Q3: 3; sync. Q5: 3), but also in the asynchronous condition (async. Q3: 2; async. Q5: 3). Comparing body ownership and experiential ownership, Q1 in both conditions was significantly lower than Q3 (sync.: Z = 3.327, *p* < 0.001, effect r = 0.403; async.: Z = 2.970, *p* = 0.003, effect r = 0.360) and lower than Q5 (sync.: Z = 2.818, *p* = 0.004, effect r = 0.342; async.: Z = 3.937, *p* < 0.001, effect r = 0.477), indicating that body ownership and experiential ownership are two distinct kinds of subjective experience. Especially, in the asynchronous condition, there was a clear contrast between a very weak sense of body ownership and a strong sense of experiential ownership.

### Experiment 2: Full body

In Experiment 2, we conducted the standard full-body experiment. Under visual manipulation, the participant watched an experimenter’s body from the adopted 1PP and received either synchronous or asynchronous tactile stimulations (Fig. 1C,D). The box charts of each question and SCR values are shown in Fig. 2C,D. The body ownership scores in the synchronous condition were significantly higher than that in the asynchronous condition (Q1: Z = 4.075, *p* < 0.001, effect r = 0.526; Q2: Z = 3.906, *p* < 0.001, effect r = 0.504), as was also true of SCR (Z = 2.376, *p* = 0.015, effect r = 0.307). Regarding experiential ownership, the medians of Q3 and Q5 were positive in both the synchronous and asynchronous conditions (sync. Q3: 2; sync. Q5: 3; async. Q3: 1.5; async. Q5: 3). Comparing questions, Q1 was significantly lower than Q3 in the asynchronous condition (Z = 2.070, *p* = 0.039, effect r = 0.267). Q1 was also significantly lower than Q5 in both conditions (sync.: Z = 3.226, *p* < 0.001, effect r = 0.416; async.: Z = 4.483, *p* < 0.001, effect r = 0.579). Again, these results indicated that experiential ownership is distinct from body ownership. They also supported that in the asynchronous condition, the participants experienced the sense of experiential ownership without the sense of body ownership.

### Cross-analysis of Experiments 1 and 2

We tested the main hypotheses by analyzing across experiments. For the first hypothesis, we conducted an analysis using the Nonparametric Longitudinal data fixed model (nparLD) by 2 × 2 × 2 factorial design. The first factor is Synchronicity (synchronous versus asynchronous, within factor). The second is Body Scope [body part (Experiment 1) versus full body (Experiment 2), between factor]. The third is Experience Type (body ownership versus experiential ownership, within factor). In the first nparLD, the comparison for the third factor is Q1 versus Q3. The main effects occurred in Synchronicity (F = 29.567, *p* < 0.001), Body scope (F = 4.357, *p* = 0.041), and Experience Type (F = 22.961, *p* < 0.001). No interaction effects were observed. In the second nparLD, we replaced Q3 with Q5 to represent experiential ownership for the third factor. The main effects occurred in both Synchronicity (F = 9.066, *p* < 0.001) and Experience Type (F = 90.283, *p* < 0.001). There was an interaction effect between Synchronicity and Experience Type (F = 23.367, *p* < 0.001). The post-hoc analysis (two-tailed Wilcoxon signed-rank test with p-value adjustment by false discovery rate method) showed that there was a significant difference between body ownership and experiential ownership both in the synchronous (*p* < 0.001) and asynchronous conditions (*p* < 0.001). Moreover, the experiential ownership scores were significantly higher than the body ownership scores in both Experiment 1 and 2. These results show that it is possible to experience experiential ownership without body ownership. This confirms our first hypothesis.

### Experiment 3: Body part, delayed measurements

**Figure 3:**
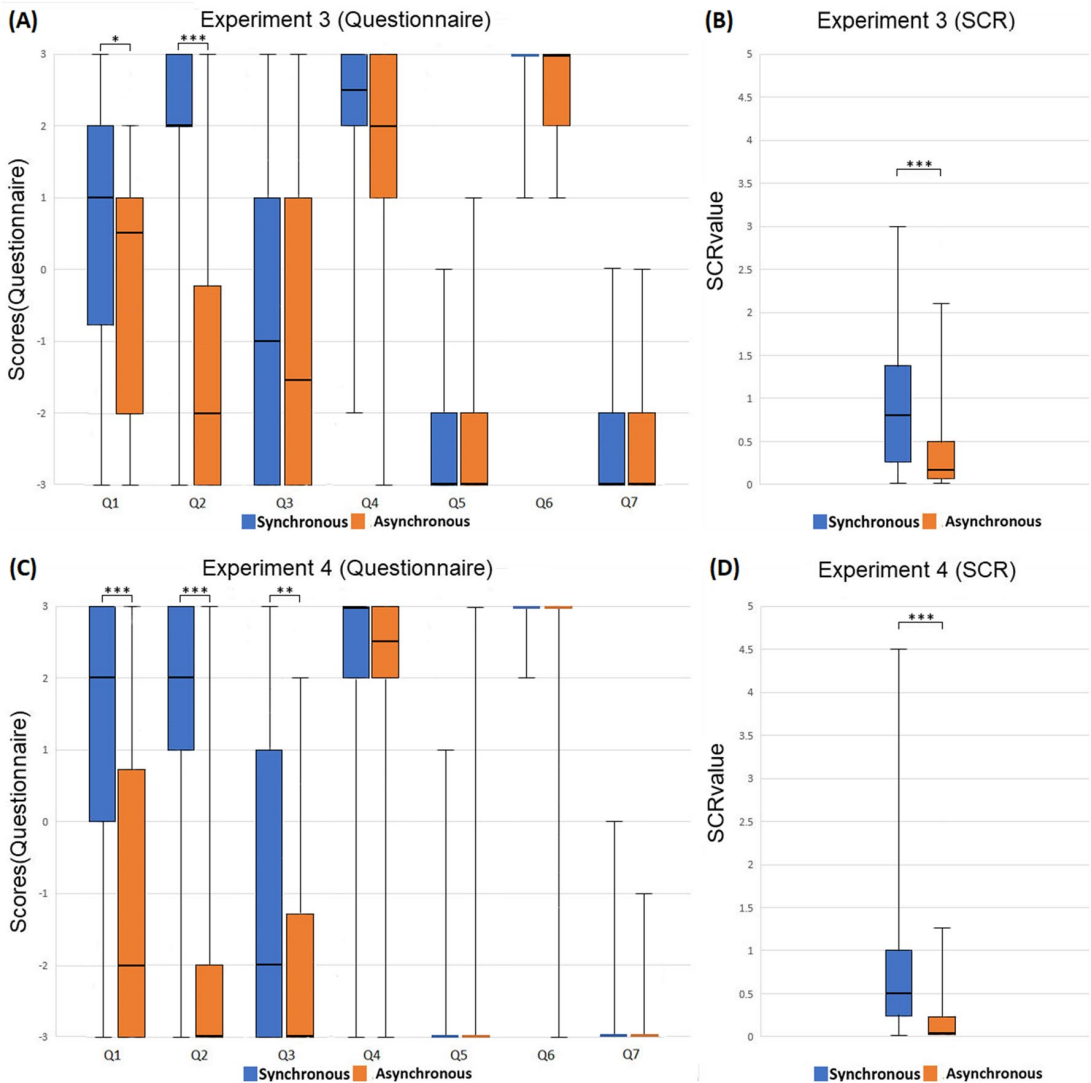
Results of Experiments 3 and 4. (**A**) The box chart of each question of Experiment 3. (**B**) The box chart of the SCR values of Experiment 3. (**C**) The box chart of each question of Experiment 4. (**D**) The box chart of the SCR values of Experiment 4. The error bars represent the maximum/minimum value. SCR value = (∆SCR in SP)/(SCR value range in RP). Significance levels: * p < 0.05; ** p < 0.01; *** p < 0.001.

The procedure of Experiment 3 was the same as that of Experiment 1, except that the SCR measurements and the questionnaire were conducted after the brushing had stopped for 5 s (Fig. 1A,B). The box charts of each question and SCR values are shown in Figs. 3A,B. We found that the body ownership scores (Q1: Z = 2.370, *p* = 0.017, effect r = 0.306; Q2: Z = 4.297, *p* < 0.001, effect r = 0.555) and SCR (Z = 3.569, *p* < 0.001, effect r = 0.461) were significantly higher in the synchronous condition, suggesting that the body ownership experience was induced and remained for a short while, even though the brushing had stopped. In contrast, the medians of the experiential ownership scores in both conditions were all negative (sync. Q3: -1; sync. Q5: -3; async. Q3: -1.5; async. Q5: -3). Q1 was significantly higher compared to Q3 in the synchronous (Z = 2.057, *p* = 0.039, effect r = 0.266) but not in the asynchronous condition (Z = 1.377, *p* = 0.177, effect r = 0.178). Q1 and Q5 were significantly different in both conditions (sync.: Z = 4.405, *p* < 0.001, effect r = 0.569; async.: Z = 4.071, *p* < 0.001, effect r = 0.526). These results indicated that, in contrast to body ownership, the sense of experiential ownership was abolished once the tactile stimulations had ended.

### Experiment 4: Full body, delayed measurements

The procedure of Experiment 4 was the same as that of Experiment 2, except that the SCR measurements and the questionnaire were conducted after the tapping had stopped for 5 s (Fig. 1C,D). The box charts of each question and SCR values are shown in Figs. 3C,D. Just like Experiment 2, the body ownership questions (Q1: Z = 3.834, *p* < 0.001, effect r = 0.495; Q2: Z = 4.350, *p* < 0.001, effect r = 0.562) and SCR (Z = 3.569, *p* < 0.001, effect r = 0.461) were significantly higher in the synchronous condition, suggesting that the sense of body ownership remained for a few seconds. In contrast, the medians of the experiential ownership scores in both conditions were all very low (sync. Q3: -2; sync. Q5: -3; async. Q3: -3; async. Q5: -3). In the synchronous condition, Q1 was significantly higher than Q3 (Z = 3.931, *p* < 0.001, effect r = 0.507) as well as Q5 (Z = 4.734, *p* < 0.001, effect r = 0.611). Again, these results showed that the sense of experiential ownership was abolished after the tactile stimulations stopped, while the sense of body ownership remained solid in the synchronous condition.

### Cross-analysis of Experiments 3 and 4

For the second hypothesis, we performed another two sets of 2 (Synchronicity, within factor) × 2 (Body Scope: Experiment 3 versus Experiment 4, between factor) × 2 (Experience Type, within factor) nparLDs. The first nparLD (third factor: Q1 versus Q3) demonstrated main effects for both Synchronicity (F = 30.870, *p* < * p* < 0.001) and Experience Type (F = 26.086, *p* < 0.001), and an interaction effect of Synchronicity and Experience Type (F = 7.853, *p* < 0.001). The post-hoc analysis showed that a significant difference of Experience Type existed in both the synchronous (*p* < 0.001) and asynchronous conditions (*p* = 0.008). The second nparLD (third factor: Q1 versus Q5) showed main effects of both Synchronicity (F = 25.301, *p* < 0.001) and Experience Type (F = 177.390, *p* < 0.001), two-way interactions between Synchronicity and Experience Type (F = 16.190, *p* < 0.001) and between Synchronicity and Body scope (F = 6.175, *p* = 0.003), and a three-way interaction (F = 8.666, *p* = 0.003). By post-hoc analysis, the Experience Type was significantly different in both the synchronous (*p* < 0.001) and asynchronous conditions (*p* < 0.001). Moreover, the experiential ownership scores were significantly lower than the body ownership scores in both Experiments 3 and 4. The overall results showed that it is possible to experience body ownership without experiential ownership. The second hypothesis is also confirmed.

### Experiment 5: SCR evidence of the experiential ownership, synchronous touch

**Figure 4:**
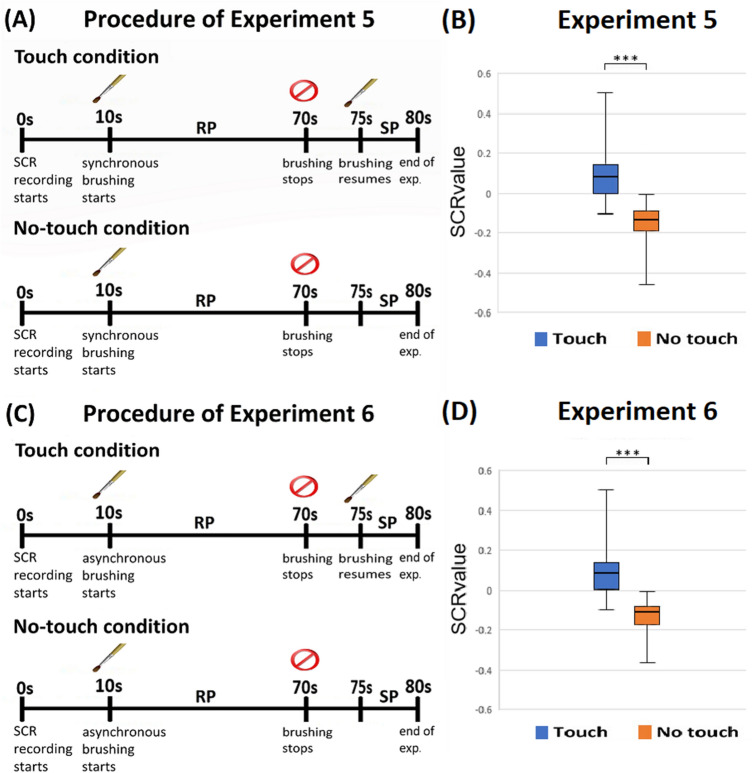
Procedures and Results of Experiments 5 and 6. (**A**) The schematic diagram of the procedures of Experiment 5. (**B**) The box chart of the SCR values of Experiment 5. (**C**) The schematic diagram of the procedures of Experiment 6. (**D**) The box chart of the SCR values of Experiment 6. The error bars represent the maximum/minimum value. SCR value = (∆SCR in SP)/(SCR value range in RP). Significance levels: * p < 0.05; ** p < 0.01; *** p < 0.001.

In Experiment 5, we used SCR to measure experiential ownership. The experimental set-up was similar to Experiments 1 and 3 (Fig. 1A,1B). The main differences were that there was no knife threat and the questionnaire was not conducted. SCR was recorded during the whole trial. At the beginning, the participants comfortably sat without receiving tactile stimulations for 10 s (Fig. 4A). At the 11th second, synchronous brushing on both the real hand and the rubber hand started and continued for 60 s. Call this the Resting Period (RP). At the 71st second, the brushing stopped for 5 s. Then, from the 76th to the 80th second, the synchronous brushing either resumed (Touch condition) or remained suspended (No-touch condition). Call this 5-s period the Stimulating Period (SP).

We observed that when tactile stimulations were provided most of the SCR signals went upwards, and that whenever there were no tactile stimulations during SP the signals always went downwards. We compared the difference in the SCR values of the Touch and No-touch conditions. The SCR value is the ratio of the delta value of SP to the value range of RP. It represents the physiological reaction in SP based on the participants’ individual differences (for more details, see the Supplementary Information). The box chart of SCR values is shown in Fig. 4B. By Wilcoxon signed-rank test, the SCR value of the Touch condition (median = 0.079) was significantly higher than that of the No-touch condition (median = − 0.139) (Z = 3.680, *p* < 0.001, effect r = 0.613), showing that during SP the participants did feel touched in the Touch condition but not in the No-touch condition. The SCR values provided physiological evidence regarding whether the participants felt touched or not during SP, which reflects the difference in experiential ownership. The results showed that the participants felt the sense of experiential ownership in the Touch condition, but not in the No-touch condition.

### Experiment 6: SCR evidence of the experiential ownership, asynchronous touch

The procedure of Experiment 6 was the same as that of Experiment 5 (Fig. 1A,B), except that the brushing was asynchronous throughout the entire experiment, including in both the Touch and No-touch conditions. (Fig. 4C). Similar to Experiment 5, we also observed that the SCR signals went upwards when tactile stimulations were provided and went downwards when tactile stimulations were not provided. The box chart of SCR values is shown in Fig. 4D. The SCR value of the Touch condition (median = 0.086) was significantly higher than the No-touch condition (median = -0.107) (Z = 3.516, *p* < 0.001, effect r = 0.622). Again, the results suggested that the sense of experiential ownership manifested only in the Touch condition.

### Cross-analysis of Experiments 5 and 6

We conducted a 2 (Synchronicity, between factor) × 2 [Touch (Touch conditions versus No-touch conditions, within factor)] nparLD to analyze all four conditions in Experiments 5 and 6. A main effect for Touch (F = 95.905, *p* < 0.001) was demonstrated. There was no interaction effect between Synchronicity and Touch. These results suggest that the participant’s experience of experiential ownership was not sensitive to whether the tactile stimulations were synchronous or asynchronous. It was affected only by whether or not they felt touched during SP.

The procedures and purpose of SCR measurements in Experiments 5 and 6 were very different from those in Experiments 1 ~4. In Experiments 1 ~ 4, SCR was measured when a knife threat was presented. It recorded the participants’ physiological responses when they felt that their own body was being threatened. According to the results (Figs. 2B,D, 3B,D), these responses depended on whether the tactile sensations were synchronous, and the statistical differences of responses provided independent evidence for the sense of body ownership. In contrast, in Experiments 5 and 6, SCR was measured without any knife threat. It revealed only whether the participants felt touched or not. As presented in Fig. 4B,D, during SP the participants’ SCR values were significantly higher when they felt touched, regardless of whether the tactile stimulations were synchronous or asynchronous. These results showed that in SP of the Touch conditions of Experiments 5 and 6 the participants felt that they were the subjects of those tactile sensations, and hence provided independent evidence for their sense of experiential ownership. In SP of the No-touch conditions, the participants did not feel the sense of experiential ownership because tactile stimulations did not occur.

## Discussion

In both the body-part and the full-body experiments, our questionnaire results showed that body ownership and experiential ownership were not the same phenomena. We found in Experiments 1 and 2 that, while body ownership was sensitive to whether the tactile manipulations were synchronous or asynchronous, experiential ownership was not. This is reasonable because, even when the tactile sensations were felt asynchronously with respect to the visual touch such that the sense of body ownership was weakened or hindered, it was still the participants *who* felt those tactile sensations. As long as the participants felt that they were touched during the experiment, they would have the sense of experiential ownership in relation to those tactile sensations, regardless of whether the stimulations were synchronous or asynchronous. In Experiments 3 and 4, we found that the sense of experiential ownership vanished as soon as the tactile manipulations stopped. This was not the case of body ownership. Since the participants watched a fake hand or someone else’s body throughout the trials, this difference could be partially explained by the role that vision plays in body ownership. While tactile sensations were necessary for both body ownership and experiential ownership, vision was not required for experiential ownership as it was for body ownership. Therefore, our study shows that it is possible for healthy subjects to experience the sense of experiential ownership without the sense of body ownership (Experiments 1 and 2), and it is possible to experience the sense of body ownership without the sense of experiential ownership (Experiments 3 and 4). We have demonstrated for the first time that body ownership and experiential ownership are empirically and doubly dissociable. The sense of experiential ownership is a genuine type of bodily self-consciousness that is different from the sense of body ownership.

Our SCR results provided independent evidence for the dissociation between body ownership and experiential ownership. Consistent with previous studies, the SCR results in Experiments 1 ~4 support the fact that the sense of body ownership is sensitive to the temporal relations between tactile sensation and visual touch. If one feels that one’s own body or body part is threatened, one’s physiological reaction will be greater than just looking at an irrelevant object being threatened^3–5^. In contrast, the SCR results of Experiments 5 and 6 served as physiological evidence for the sense of experiential ownership. On the one hand, the results showed that the participants did feel touched on their hand when the brushing occurred during SP. On the other hand, if the brushing did not take place during SP, then the participants felt no tactile sensations, and the SCR measurements showed no physiological response. Hence, the significant differences between the Touch and the No-touch conditions reflected differences in whether the participants had the sense of experiential ownership during SP. More importantly, this pattern was observed not only under synchronous manipulations but also under asynchronous manipulations. That is, the participants’ sense of experiential ownership was *not* sensitive to the temporal relations between tactile sensation and visual touch. This feature of experiential ownership was very different from the case of body ownership, and the SCR results that suggested it accorded nicely with what we observed in the questionnaire data.

Together, the questionnaire data and the SCR results strongly support that experiential ownership is genuinely distinct and empirically dissociable from body ownership. These findings are important because experiential ownership is a ubiquitous phenomenon, and yet is almost neglected by the scientific community. It is ubiquitous because for every conscious experience there is a unique subject who experiences it. From the first-person perspective, when I have a conscious experience, say, a tactile sensation, I feel not only that my body is touched, but also that it is *me* who is touched. Given the ubiquity and hence the importance of this phenomenon and compared with the huge size of literature on body ownership, it is surprising that empirical research on experiential ownership is scarce. Our study has contributed to remedying this situation.

However, there are two limitations in our study. First, in this study we focused on the sense of experiential ownership embedded in tactile sensation. Instead of measuring experiential ownership directly, it was measured via manipulations of tactile sensation. We did not manage to experimentally disentangle the experiential ownership of touch from mere tactile sensation. From our perspective, this limitation in fact reflects the special nature of the relationship between experiential ownership and tactile sensation. That is, every tactile sensation is constitutively associated with a sense of experiential ownership. This is because every tactile sensation is necessarily associated with a subject who experiences it, and the subject is the one who has the sense of experiential ownership of that sensation. Hence, although experiential ownership and tactile sensation are not the same thing, they are empirically inseparable. Based on this observation, we made a fundamental assumption that the sense of experiential ownership is a constitutive component of tactile perception. On this view, there is no way to measure experiential ownership in isolation from tactile sensation. Rather, the presence/absence of tactile sensation can serve as a reliable indicator of the presence/absence of its experiential ownership. The second limitation of our study is that, at this stage, the fundamental assumption just mentioned remains a philosophical one. Although this assumption has strong support from philosophical literature (cf.^28,29,31,40–43^) and we think it is correct, we concede that it would be much better if this assumption can gain support from empirical study. This will require further interdisciplinary research.

We will now address two possible objections. (1) Since we divided body ownership and experiential ownership into different questionnaire statements, one possible worry is that an expectation effect could exist to the detriment of our interpretation. But notice that, although we formulated body ownership and experiential ownership as different statements, there was no assumption as to how the participants might respond to them. They are at most conceptually distinct in our questionnaires. Whether the participants would feel them to be different can be ascertained only by empirical measurements.

(2) Our main evidence for the double dissociation between body ownership and experiential ownership comes from the questionnaire data and statistics. As some phenomenologists would argue, in answering the questionnaires the participants’ subjective experiences became objects of their reflection. Therefore, the data only revealed the participants’ cognitive and reflective judgments, rather than their subjective *pre-reflective* experiences^28,29,31,44^. The double dissociation that we demonstrated is only at the reflective or cognitive level, not at the subjective and pre-reflective level. This could not be enough to establish the claim that body ownership and experiential ownership are two different types of bodily self-consciousness.

We disagree. First, all of the participants in our experiments were healthy subjects. In our set-ups, they continued to receive tactile stimulations while answering the questionnaires. There is no compelling reason showing that there exists a gap between the participants’ judgments in the questionnaires and their pre-reflective experiences. If their cognitive responses exhibited the distinction between body ownership and experiential ownership, this could well indicate that they are two different kinds of subjective experience. Second, as one major phenomenological philosopher says, “Reflection is constrained by what is pre-reflectively lived through. It is answerable to experiential facts and is not constitutively self-fulfilling. To deny that the reflective self-ascription of beliefs is based on any experiential evidence whatsoever is implausible”^29^. We welcome this remark, which in effect impairs the objection because it suggests that the participants’ cognitive judgments were constrained by, and hence could reveal, their subjective pre-reflective experiences. Finally, our questionnaire results were supported by SCR measurements. It is widely recognized that SCR cannot be mentally controlled by the participants, i.e., it cannot be affected by cognitive effort at the reflective level. Therefore, we think that the best explanation for the results presented in this study is that the sense of body ownership and the sense of experiential ownership are empirically and doubly dissociable.

## Materials and methods

### Participants

We recruited 158 healthy volunteers for a total of six experiments (Table 1). Informed consent to participate was obtained from all the participants. The persons whose body/body part shown in Fig. 1 gave their written informed consent to publish. All experiments were conducted in accordance with the Declaration of Helsinki. This study was performed in accordance with the regulations of, and was approved by, the Research Ethics Committee of National Taiwan University (NTU-REC: 201807HS009).

### Materials

In this study, we used a stereo camera (Sony HDR-TD20V) and a head-mounted display (HMD, VISIONHMD BIDEYES-H1) in Experiments 2 and 4. To record participants' skin conductance responses (SCR), we used a Data Acquisition Unit Biopac MP35 (Goleta, USA). For the questionnaires, we used a Likert scale from “strongly disagree” (− 3) to “strongly agree” (+ 3). The questions were randomly distributed and divided into four categories: body ownership, experiential ownership, supporting questions, and control questions. The questionnaires were in Chinese when presented to the participants. Table 1 in the main text presents the English translations.

### Procedures

#### Body-part Experiments (Experiment 1 and 3)

Experiment 1 is the paradigmatic RHI setting. The participant placed his/her right hand on a desk and the hand was blocked from view, so that the participant saw a rubber hand from the 1PP. An experimenter used paintbrushes to brush both the participant’s and the rubber hand for 60 s (the frequency was approximately once every two seconds) either synchronously or asynchronously, followed by a knife threat to measure SCR. Then the participant orally answered the questionnaire. The brushing continued while another experimenter conducted SCR measurements and the questionnaire. To present a knife threat, we displayed the knife in the participant’s line of sight for one second to make sure that he/she could see it, and then pretended to cut the rubber hand from left to right. The SCR values that we analyzed refer to the ratio of delta value in the period of threat (SP) to the value range in the period before the knife was present (RP). In short, SCR value = (raw delta value in SP)/(raw value range in RP). For details, please see Supplementary Information. In Experiment 3, the knife threat, the SCR measurements and the questionnaire were conducted after the brushing had stopped for 5 s.

#### Full-body Experiments (Experiments 2 and 4)

The participant wore a laboratory coat and put on an HMD connected to a stereo camera filming a torso and two legs in real time. What the participant saw via the HMD was actually the experimenter’s torso and legs from the adopted 1PP. Then a second experimenter used wooden rods to tap on both the participant’s and the first experimenter’s left legs either synchronously or asynchronously for 60 s, followed by a knife threat to measure SCR. Then the participant orally answered the questionnaire. The tapping continued while another experimenter conducted SCR measurements and the questionnaire. Similarly, the knife came into the participant’s view for one second first and then was used to pretend to cut the seen body from left to right. Again, SCR values = (raw delta value in SP)/(raw value range in RP). In Experiment 4, the knife threat, the SCR measurements and the questionnaire were conducted after the tapping had stopped for 5 s.

#### Physiological evidence of the experiential ownership (Experiments 5 and 6)

The set-ups of Experiments 5 and 6 were similar to Experiment 1, a typical RHI setting, except that there was no knife threat. The participant placed his/her right hand on a desk and the hand was blocked from view, so that the participant saw a rubber hand from the 1PP. For the schematic diagram of the procedures, please see Fig. 4A,C. SCR was recorded from the beginning of the experiment. At the 11th second, an experimenter used paintbrushes to brush both the participant’s and the rubber hand either synchronously (Experiment 5) or asynchronously (Experiment 6) for 60 s (i.e., until the end of the 70th second). This 60-s period was Resting Period (RP), during which we recorded each participant’s SCR value range as the basis of individual physiological reaction. The brushing stopped from the 71st to the 75th second. Then the Stimulating Period (SP) started from the 76th to the 80th second. In the Touch conditions, the synchronous (Experiment 5) or asynchronous (Experiment 6) brushing resumed during these 5 s. In the No-touch conditions of both experiments, the brushing remained stopped. We recorded the delta values of SCR during SP to compare the physiological responses between the Touch and the No-touch conditions by using the following formula: SCR value = (raw delta value in SP)/(raw value range in RP). (Notice that the SP and RP here are different from those in Experiments 1 ~4).

The Shapiro–Wilk tests showed that the questionnaire scores and the SCR data were not normally distributed. To compare between the synchronous and asynchronous conditions within each experiment, we used non-parametric Wilcoxon signed-rank tests (two-tailed). In the cross-experiment analyses, we chose nonparametric longitudinal data fixed models (nparLD) for factorial analysis, followed by Wilcoxon signed rank tests for the post-hoc multiple comparisons. For more details about statistics, please see Supplementary Information.

## Supplementary Information

Supplementary Information 1.

Supplementary Information 2.

Supplementary Information 3.
