# Supplementary figures and images for: Experiential ownership and body ownership are different phenomena
Source: Sci Rep. 2021 May 19;11:10602. doi: 10.1038/s41598-021-90014-y (PMC8134432; doi:10.1038/s41598-021-90014-y)

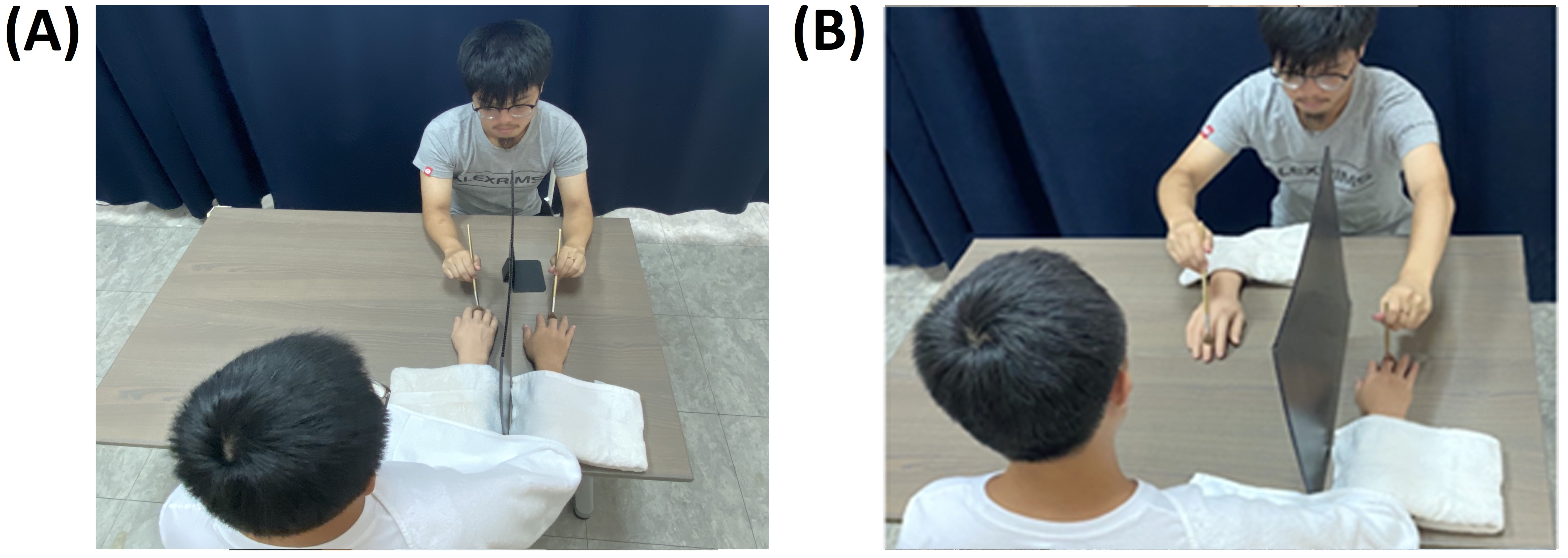

Supplement: Supplementary file 1 — Supplementary Information 1. [file 41598_2021_90014_MOESM1_ESM.png]

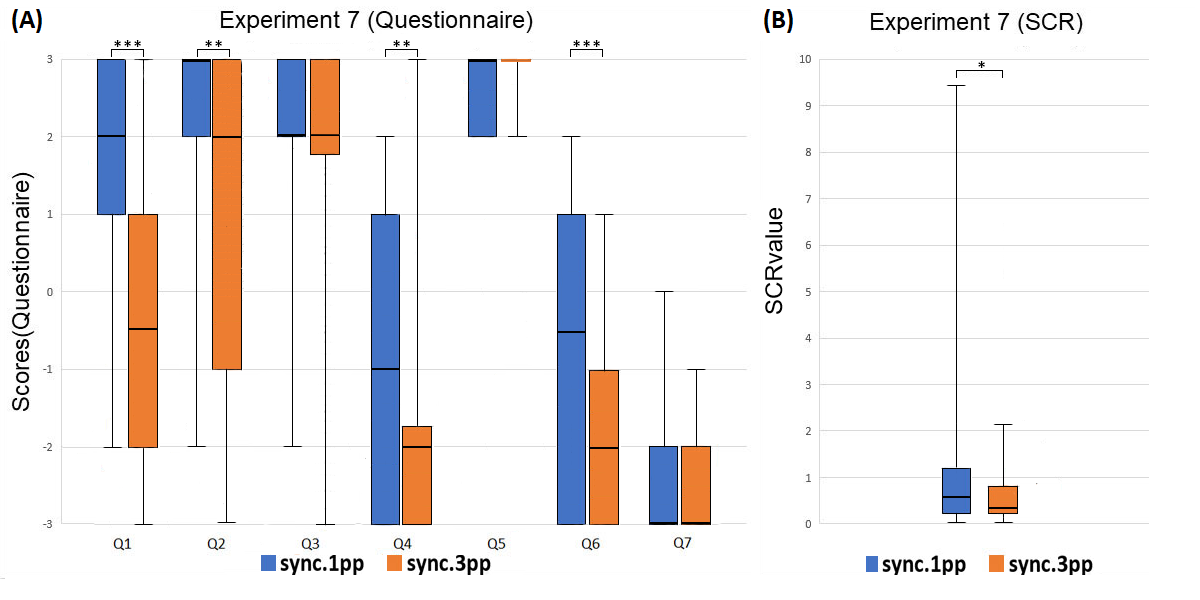

Supplement: Supplementary file 2 — Supplementary Information 2. [file 41598_2021_90014_MOESM2_ESM.png]
