# Supplementary material for: Experiential ownership and body ownership are different phenomena
Source: Sci Rep. 2021 May 19;11:10602. doi: 10.1038/s41598-021-90014-y (PMC8134432; doi:10.1038/s41598-021-90014-y)
Supplement: Supplementary file 3 — Supplementary Information 3. [file 41598_2021_90014_MOESM3_ESM.docx]

**Experiential ownership and body ownership**

**are different phenomena**

**Caleb Liang^1, 2^, Wen-Hsiang Lin^3^, Tai-Yuan Chang^1, 4^, Chi-Hong Chen^1, 5^,**

**Chen-Wei Wu^1^, Wen-Yeo Chen^2^, Hsu-Chia Huang^2^, Yen-Tung Lee^6, 7, *^**

**^1^** Department of Philosophy, National Taiwan University, Taiwan

**^2^** Graduate Institute of Brain and Mind Sciences, National Taiwan University, Taiwan

**^3^** Graduate Institute of Digital Learning and Education, National Taiwan University of Science and Technology, Taiwan

**^4^** Department of Law, National Taiwan University, Taiwan

**^5^** Department of Economics, National Taiwan University, Taiwan

**^6^** Department of Philosophy, Western University, Canada

**^7^** Rotman Institute of Philosophy, Western University, Canada

* Corresponding author: Yen-Tung Lee

Email: ylee734@uwo.ca

Keywords: body ownership, experiential ownership, self-consciousness, bodily illusion

**Supplementary Information**

The Supporting Information here consists of three parts: (1) Supplementary descriptions of Materials and Methods; (2) Additional data of Experiments 1 ~ 4 and remarks; (3) A supporting experiment that involved comparisons of the first-person perspective (1PP) and the third-person perspective (3PP).

**Materials and Methods**

Informed consent to participate was obtained from all the participants. The persons whose body/body part shown in SI Figure 1 gave their written informed consent to publish. All experiments were conducted in accordance with the Declaration of Helsinki. This study was performed in accordance with the regulations of, and was approved by, the Research Ethics Committee of National Taiwan University (NTU-REC: 201807HS009).

**Procedure**

In all of our experiments, each volunteer participated in both the synchronous and the asynchronous conditions, the order of which was randomized. For the questionnaire, the order of the questions was also randomized. To mask any sounds made by the operation, all participants wore earphones, and white noise was played during the experiment.

**Skin Conductance Responses (SCR)**

SCR has been considered by previous studies as reliable physiological evidence for the subjective experience of body ownership ^[1,2,3,4,5,6,7]^. This is due to the fact that the physiological reaction to a knife threat is controlled by the automatic nervous system and not influenced by one’s reflection. If one feels a certain body as one’s own, the reaction will be greater when the body is threatened. We adopted this paradigm in Experiments 1 ~ 4. To measure SCR, we attached two single-use foam electrodes (Covidien, Inc., Mansfield, USA) to the inner side of the participant’s left palm. One was placed 2 cm below the thumb and the other 4 cm below the little finger. The wires were carefully placed under the participant’s arm so that neither the electrodes nor the wires would be seen by the participant. To analyze SCR, the sampling rate was set at 200 samples per second, and the Hardware Filter was set to 20 KHz Lowpass. Data were analyzed with the Biopac software AcqKnowledge v. 3.7.7. We followed the standard procedure and identified the amplitude of SCR as the difference between the maximal and minimal values of the responses within 5 seconds of the threat (call this time range the Stimulating Period) ^[8]^.

To avoid large variations in SCR values due to extraneous individual differences rather than experimental manipulations, we adopted Lykken et al.’s range correction to analyze the raw data ^[9]^. By this method, the amplitude of one’s SCR response depends not only on the difference between the peak value and the lowest value during the Stimulating Period (SP), but also on the ratio of value range in one’s SP to the Resting Period (RP, the period before the knife was present). Taking this into account helps to avoid the problem that a seemingly high peak value might not really represent a high physiological response when one’s SCR value in RP is much higher than that of other subjects. We therefore used a formula that transforms the raw data in the following way: SCR value = (raw delta value in SP) / (raw value range in RP). Then we analyzed the SCR values by Wilcoxon signed-rank tests and nonparametric longitudinal data fixed models. For technical discussions of this method, please see Dawson et al. (2006, p. 166). ^[8]^

The aim of the SCR measurements in Experiments 5 and 6 was to measure the sense of experiential ownership, not the sense of body ownership. We used the same formula to translate the raw values [SCR value = (raw delta value in SP) / (raw value range in RP)], but due to the different procedures in Experiments 5 and 6, the meanings of RP and SP changed accordingly. There was no knife threat in these two experiments. In Experiments 5 and 6, RP referred to the period during which the participants received tactile stimulations (from the 11^th^ to the 70^th^ second). RP was followed by a 5-second intermission from the 71^st^ to the 75^th^ second. SP referred to the period from the 76^th^ to the 80^th^ second, during which the brushing resumed in the Touch conditions but not in the No-touch conditions of Experiments 5 and 6. Our main target was to measure the SCR response in SP, which represented the physiological response when the brushing resumed, as compared to the No-touch conditions. Since SP lasted for only 5 seconds, the participant’s SCR response was assumed to be a single reaction ^[8]^. In the No-touch conditions of Experiments 5 and 6, since there were no tactile stimulations during SP, we expected that there would be no physiological reactions. Typically, the SCR signals would go downwards when there were no reactions. In order to differentiate between the presence and absence of reactions, the SCR responses were recorded in the following way: If the reaction went upwards, we recorded the positive delta value. If the reaction went downwards, we recorded the delta value as negative.

**Questionnaire**

The seven statements in each questionnaire have different functions. **Q1** (Exp. 1&2: “It felt as if I was looking at my hand/body”; Exp. 3&4: “Right now, it feels as if I am looking at my hand/body”) was a paradigmatic body ownership question. It measured whether the participants felt the body part or the full body that they were watching as their own. **Q2** (Exp. 1~4: “The touch that I felt was caused by the paintbrush/stick in front of me”) was a touch referral question that is pervasively considered as a support to body ownership ^[10,11]^.

**Q3** and **Q5** were the main experiential ownership questions. **Q3** (Exp. 1&2: “During the experiment it was me who felt touched”; Exp. 3&4: “Right now, it seems that it is me who is feeling touched”) specifically targeted the *who*-aspect of participants’ subjective experience, asking whether they felt themselves to be the subject of the experience. **Q5** (Exp. 1&2: “I felt that I was being touched during the experiment”; Exp. 3&4: “I am feeling touched right now”) was another experiential ownership question. Although **Q5** does not contain the word “who,” it has the advantage of focusing on touch. In case some participants understood **Q3** merely via vision instead of tactile sensation, this potential confounding factor would be nullified by **Q5**. Thus, **Q3** and **Q5** are complementary to each other.

**Q4** and **Q6** were contrast questions to **Q3** and **Q5**, respectively. **Q4** (Exp. 1&2: “During the experiment it was me who felt pain/tickled”; Exp. 3&4: “It seems that it was me who felt touched a moment ago”) was contrasting to **Q3** by type of sensation (Exp. 1&2) or time (Exp. 3&4). The goal was to make the measurements of experiential ownership by **Q3** more precise. In Experiments 1 and 2, **Q4** helped to avoid the possibility that the participants might associate experiential ownership with another type of experience (e.g. pain) not controlled by the experiment such that their positive answer of **Q3** did not represent their sense of experiential ownership due to the tactile stimulations. We predicted that **Q4** would be significantly lower than **Q3**. This was confirmed by our data presented in the next section, which made sure that the participants answered **Q3** according to their tactile experience.

In the first 60 seconds of Experiments 3 and 4, we induced the sense of experiential ownership by tactile stimulations, and then stopped the touching to abolish it. The role of **Q4** was to consolidate the measurements by **Q3**. It eliminated the possibility that in some cases the sense of experiential ownership was not induced in the first 60 seconds at all, such that we would not be completely sure that the sense of experiential ownership was really abolished by ending the tactile stimulations. We predicted that **Q4** would be significantly higher than **Q3** in Experiments 3 and 4. Again, this was confirmed by our data presented in the next section. **Q6** (Exp. 1&2: “I felt that I was being hit/tickled during the experiment”; Exp. 3&4: “I felt that I was touched a moment ago”) was designed for the same reasons in relation to **Q5** (see the next section). Finally, **Q7** (Exp. 1~4: “It felt as if the hand/body in front of me gradually became a flower”) was a control question, precluding the possibility that the participants did not answer the questionnaire according to their experience. If a participant answered **Q7** positively, that would be regarded as failing the control and his/her data would be excluded from the following analyses.

**Data analysis**

The Shapiro-Wilk tests showed that the questionnaire scores and the SCR data were not normally distributed. So, we used non-parametric statistics. For the comparisons between the synchronous and asynchronous conditions in each experiment, we used two-tailed Wilcoxon signed-rank tests to analyze the differences of each item and the SCR data. For the cross-experiment analysis, we conducted nonparametric longitudinal data fixed models (nparLD) using R (version 4.0.4). The nparLD method is developed by Noguchi and colleagues as an R package to analyze higher-way factorial layouts of data^[12,13]^. Its theoretical ground is based on Brunner and Puri ^[14]^ and Puri et al. ^[15]^. Specifically, we adopted the ANOVA-type analysis (ATS) of nparLD for our repeated-measures designs ^[12]^. Since ATS becomes too conservative when testing an effect with a finite denominator degree of freedom with sub-plot factors involved, we modified the degree of freedom for all the relevant F-values by F_(f, ∞)_ rather than F_(f, f0)_ ^[13]^. We did four sets of 2 (Synchronicity, within factor) × 2 (Body Scope: Experiment 3 vs. Experiment 4, between factor) × 2 (Experience Type, within factor) nparLD analyses. Two of which were presented in Cross-analysis of Experiments 1 and 2, and the rest in Cross-analysis of Experiments 3 and 4. Moreover, we did an extra 2 (Synchronicity, between factor) × 2 [Touch (Touch conditions vs. No-touch conditions, within factor)] nparLD, which is presented in Cross-analysis of Experiments 5 and 6. Overall, five nparLD analyses were conducted. Finally, for the post-hoc multiple comparisons, we programed R software to conduct two-tailed Wilcoxon signed rank tests with p-value adjusted by the false discovery rate method. We focused on the factor Experience Type and its relationship with other factors, all of which with significant results are presented in the manuscript.

**Additional Data of Experiments 1 ~ 4**

The tables in this section include the median values and interquartile ranges (IQR), and p-values by two-tailed Wilcoxon signed-rank test.

**Experiment 1: Body part**

|  | **Q1** | **Q2** | **Q3** | **Q4** | **Q5** | **Q6** | **Q7** | **SCR** |
| --- | --- | --- | --- | --- | --- | --- | --- | --- |
| **sync.** | **2 (1~3)** | **2 (-0.75~3)** | **3 (2~3)** | **-3 (-3~-1)** | **3 (3~3)** | **-3 (-3~-1)** | **-3 (-3~-2)** | **0.52 (0.25~0.83)** |
| **async.** | **1(-2.75~2)** | **-3 (-3~-1.25)** | **2 (2~3)** | **-2 (-3~0)** | **3 (3~3)** | **-2 (-3~-1)** | **-3 (-3~-2)** | **0.29 (0.14~0.61)** |
| **p-value** | **<0.001** | **<0.001** | **0.057** | **0.178** | **0.695** | **0.946** | **0.846** | **0.017** |

Comparisons of contrast questions: Sync.: **Q3** vs. **Q4** Z = 4.992, p < 0.001, effect r = 0.605; **Q5** vs. **Q6** Z = 4.983, p < 0.001, effect r = 0.604. Async.: **Q3** vs. **Q4** Z = 4.235, p < 0.001, effect r = 0.514; **Q5** vs. **Q6** Z = 4.901, p < 0.001, effect r = 0.594.

Two remarks: (1) The median value of **Q1** in the asynchronous condition was slightly positive rather than negative. This was because the rubber hand we used in the experiment was a customized product by an assistive device company (Taiwan OnP Co.), which was much more realistic than those used in previous literature ^[16]^. This value was significantly lower than that of the synchronous condition. It at least showed that, overall, the participants were uncertain about their experience of body ownership. Since the sense of body ownership was positively induced in the synchronous condition but not in the asynchronous condition, it remained the case that a single dissociation between body ownership and experiential ownership occurred in the asynchronous condition.

(2) We predicted that high median values of experiential ownership would be observed in both the synchronous and asynchronous conditions. Because, as long as the participants felt that they had been touched during the experiment, they would have the sense of experiential ownership with regard to those tactile sensations, regardless of whether the tactile stimulations were synchronous or asynchronous. This prediction was confirmed by the data presented above. In the results of **Q3**, there was a significant difference between the synchronous and asynchronous conditions. Since the median values of **Q3** in both conditions were very high, and the median values of **Q5** were even higher and did not show a similar significance, the significant difference in **Q3** did not violate our prediction.

**Experiment 2: Full body**

|  | **Q1** | **Q2** | **Q3** | **Q4** | **Q5** | **Q6** | **Q7** | **SCR** |
| --- | --- | --- | --- | --- | --- | --- | --- | --- |
| **sync.** | **2 (1~2.75)** | **2 (1~3)** | **2 (2~3)** | **-2 (-3~0)** | **3 (2~3)** | **-3 (-3~-1)** | **-3 (-3~-2)** | **0.18 (0.08~0.53)** |
| **async.** | **-1 (-2~1)** | **-2 (-3~-1)** | **1.5 (-2~3)** | **-3 (-2.5~0)** | **3 (2~3)** | **-3 (-3~-2)** | **-3 (-2.25~-3)** | **0.09 (0.03~0.30)** |
| **p-value** | **<0.001** | **<0.001** | **0.004** | **1** | **0.519** | **0.102** | **0.652** | **0.016** |

The median of **Q3** in the asynchronous condition was consistent with our prediction, but it was not as high as we expected. Fortunately, this was compensated by other related data. For example, the median of **Q5** in the asynchronous condition was very high, and the contrasts between **Q3** and **Q4**, and between **Q5** and **Q6**, were quite strong (Sync.: **Q3** vs. **Q4** Z = 4.493, p < 0.001, effect r = 0.580; **Q5** vs. **Q6** Z = 4.737, p < 0.001, effect r = 0.612. Async.: **Q3** vs. **Q4** Z = 2.972, p = 0.002, effect r = 0.384; **Q5** vs. **Q6** Z = 4.861, p < 0.001, effect r = 0.628). Also, as presented in the main text, **Q1** was significantly lower than **Q3** in the same condition (p = 0.039).

**Experiment 3: Body part, delayed measurements**

|  | **Q1** | **Q2** | **Q3** | **Q4** | **Q5** | **Q6** | **Q7** | **SCR** |
| --- | --- | --- | --- | --- | --- | --- | --- | --- |
| **sync.** | **1 (-0.75~2)** | **2 (2~3)** | **-1 (-3~1)** | **2.5 (2~3)** | **-3 (-3~-2)** | **3 (3~3)** | **-3 (-3~-2)** | **0.77 (0.33~1.35)** |
| **async.** | **0.5 (-2~1)** | **-2 (-3~-0.25)** | **-1.5 (-3~1)** | **2 (1~3)** | **-3 (-3~-2)** | **3 (2~3)** | **-3 (-3~-2)** | **0.24 (0.14~0.53)** |
| **p-value** | **0.017** | **<0.001** | **0.470** | **0.058** | **0.578** | **0.156** | **1** | **<0.001** |

Comparisons of contrast questions: Sync.: **Q3** vs. **Q4** Z = 4.187, p < 0.001, effect r = 0.540; **Q5** vs. **Q6** Z = 4.887, p < 0.001, effect r = 0.631. Async.: **Q3** vs. **Q4** Z = 3.875, p < 0.001, effect r = 0.500; **Q5** vs. **Q6** Z = 4.912, p < 0.001, effect r = 0.634.

**Experiment 4: Full body, delayed measurements**

|  | **Q1** | **Q2** | **Q3** | **Q4** | **Q5** | **Q6** | **Q7** | **SCR** |
| --- | --- | --- | --- | --- | --- | --- | --- | --- |
| **sync.** | **2 (0~3)** | **2 (1~3)** | **-2 (-3~1)** | **3 (2~3)** | **-3 (-3~-3)** | **3 (3~3)** | **-3(-3~-3)** | **0.52 (0.32~0.96)** |
| **async.** | **-2 (-3~0.75)** | **-3 (-3~-2)** | **-3 (-3~-1.25)** | **2.5 (2~3)** | **-3 (-3~-3)** | **3 (3~3)** | **-3 (-3~-3)** | **0.10 (0.06~0.30)** |
| **p-value** | **<0.001** | **<0.001** | **0.005** | **0.821** | **0.813** | **0.438** | **0.500** | **<0.001** |

Comparisons of contrast questions: Sync.: **Q3** vs. **Q4** Z = 3.876, p < 0.001, effect r = 0.500; **Q5** vs. **Q6** Z = 5.062, p < 0.001, effect r = 0.654. Async.: **Q3** vs. **Q4** Z = 4.396, p < 0.001, effect r = 0.568; **Q5** vs. **Q6** Z = 4.874, p < 0.001, effect r = 0.629.

**Experiment 7: body part, sync. 1PP vs. sync. 3PP**

Finally, we report a supplementary experiment, Experiment 7, to test our first hypothesis by comparing 1PP and 3PP. 36 volunteers were recruited in this experiment (age mean: 21.8±2.5; 13 males). All participants gave written consent prior to the experiment, which was approved by the Research Ethics Committee of National Taiwan University (NTU-REC: 201807HS009).

Previous studies have shown that perspective is a crucial factor for body ownership. According to Costantini and Haggard ^[17]^, a necessary condition of RHI is that the felt posture of the real hand matches the posture of the seen hand, i.e., the participant must look at the rubber hand from the 1PP. Tsakiris ^[18]^ also includes such a factor as an integral component of his RHI model, arguing that a discrepancy in the posture prevents the induction of body ownership experience. Our Experiment 7 consists of two conditions: the synchronous 1PP condition vs. the synchronous 3PP condition. Each subject participated in both conditions and the order was randomized. The participant placed his/her right hand on a desk and was blocked from view and saw a rubber hand either from the 1PP or from the 3PP (**SI Figure 1**). In the 1PP condition, the participants saw the rubber hand from the 1PP, while in the 3PP condition they saw it stretching out from the opposite side. An experimenter used paintbrushes to brush both the participant’s and the rubber hand for 60 seconds (the frequency was approximately once every two seconds), followed by a knife threat to measure SCR. In both conditions, the tactile stimulations were synchronous. Then the participant orally answered the questionnaire. The brushing continued while another experimenter conducted SCR measurements and the questionnaire.

| 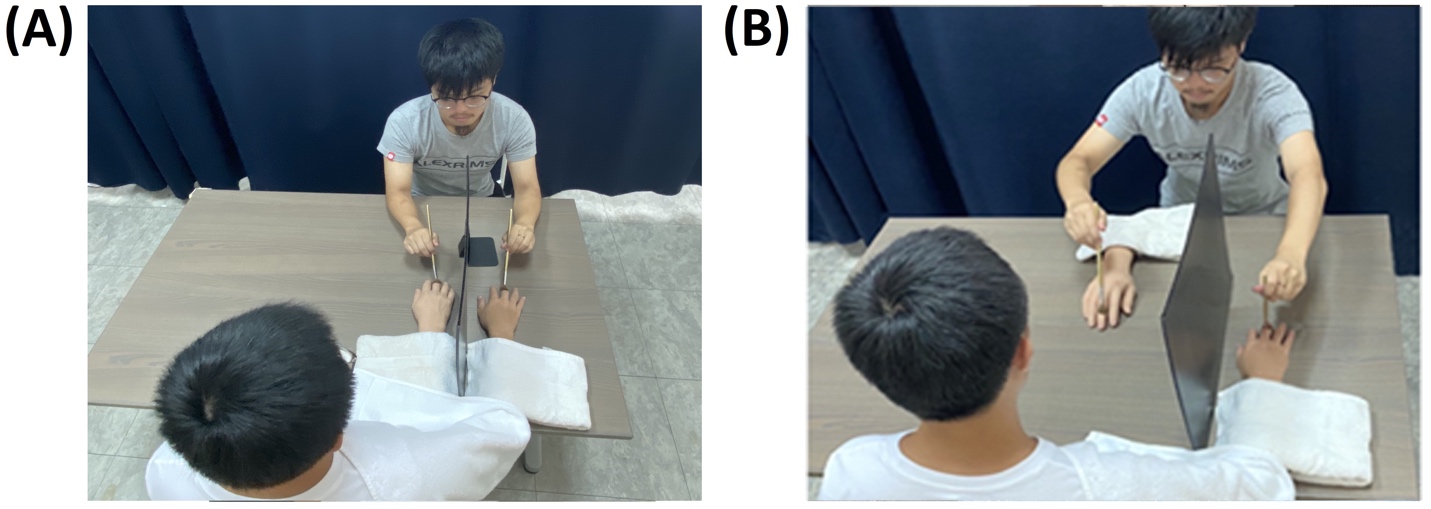 |
| --- |
| **SI Figure 1.** **Setting of Experiment 7**  **(A)** The setting of the 1PP condition. The participant placed his/her right hand on a desk and the hand was blocked from view. What the participant saw was a rubber hand (the left one) from the 1PP. The experimenter brushed both hands synchronously. **(B)** The setting of the 3PP condition. The participant placed his/her right hand on a desk and the hand was blocked from view. What the participant saw was a rubber hand (the left one) stretching from the opposite side. The experimenter brushed both hands synchronously. |

We predicted that, first, the participants would experience the RHI only in the 1PP condition. Second, we predicted that high medians of experiential ownership would be observed in both the 1PP and 3PP conditions. Because, as long as the participants felt tactile sensations during the experiment, the sense of experiential ownership would be induced with regard to those sensations regardless of whether the rubber hand was seen from the 1PP or from the 3PP. Our third prediction was that the results of experiential ownership in the 3PP condition would be significantly higher than that of body ownership. If these three predictions are confirmed, they can support our first hypothesis mentioned in the main text that it is possible for a subject to experience experiential ownership without body ownership. Here are the results, including the medians, IQRs and p-values by two-tailed Wilcoxon signed-rank test:

|  | **Q1** | **Q2** | **Q3** | **Q4** | **Q5** | **Q6** | **Q7** | **SCR** |
| --- | --- | --- | --- | --- | --- | --- | --- | --- |
| **1PP** | **2 (1~3)** | **3 (2~3)** | **2 (2~3)** | **-1 (-3~1)** | **3 (2~3)** | **-0.5 (-3~1)** | **-3 (-3~-2)** | **0.62 (0.34~1.18)** |
| **3PP** | **-0.5 (-2~1)** | **2 (-1~3)** | **2 (1.75~3)** | **-2 (-3~-1.75)** | **3 (3~3)** | **-2 (-3~-1)** | **-3 (-3~-2)** | **0.43 (0.25~0.83)** |
| **p-value** | **<0.001** | **0.001** | **0.508** | **0.002** | **0.426** | **<0.001** | **0.496** | **0.024** |

All of these predictions were confirmed by the data. The box chart of each question is shown in **SI Figure 2**. Both the questions of body ownership (**Q1**: Z = 4.240, p < 0.001, effect r = 0.500; **Q2**: Z = 3.070, p = 0.001, effect r = 0.362) and SCR (Z = 2.247, p = 0.024, effect r = 0.265) showed significant differences with respect to hand orientation. The sense of body ownership was induced only in the 1PP condition. This was consistent with previous studies ^[18, 19]^, suggesting that hand orientation is a crucial factor to body ownership experience. In contrast, the experiential ownership questions **Q3** and **Q5** did not show similar significances. The medians of **Q3** and **Q5** were very high in both the 1PP and the 3PP conditions. This indicates that, contrary to the sense of body ownership, the sense of experiential ownership was not sensitive to hand orientation. Finally, **Q1** was significantly lower than **Q5** in both conditions (1PP: Z = 3.280, p < 0.001, effect r = 0.387; 3PP: Z = 5.039, p < 0.001, effect r = 0.594). **Q1** was also lower than **Q3** in the 3PP condition (Z = 3.915, p < 0.001, effect r = 0.461). The contrasts between **Q3** and **Q4**, and between **Q5** and **Q6**, were also quite strong (1PP: **Q3** vs. **Q4** Z = 4.880, p < 0.001, effect r = 0.575; **Q5** vs. **Q6** Z = 5.119, p < 0.001, effect r = 0.603. 3PP: **Q3** vs. **Q4** Z = 5.048, p < 0.001, effect r = 0.595; **Q5** vs. **Q6** Z = 5.287, p < 0.001, effect r = 0.623). Overall, these data showed that the sense of body ownership and the sense of experiential ownership are distinct types of subjective experience. This experiment provides additional support for our first hypothesis that it is possible for participants to experience experiential ownership without body ownership.

| 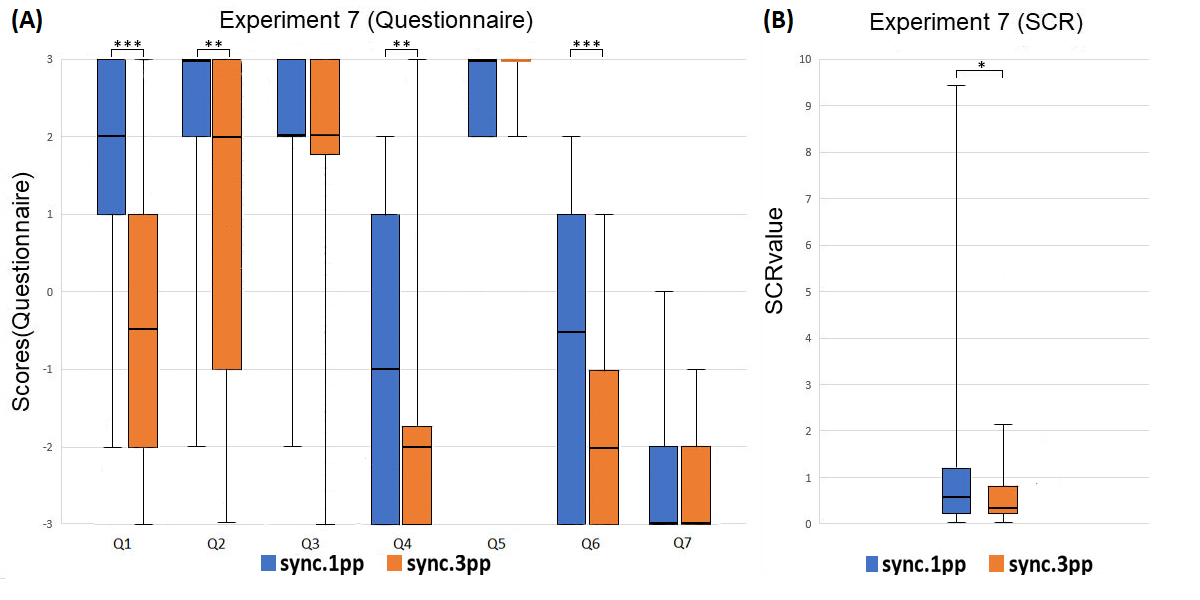 |
| --- |
| **SI Figure 2.** **Results of Experiment 7**  **(A)** The box chart of each question in both conditions. **(B)** The box chart of SCR values in both conditions. The error bars represent the maximum/minimum value. SCR value = (∆SCR in SP) / (SCR value range in RP). Significance levels: *p < 0.05; **p < 0.01; ***p < 0.001. |

**References**

1. Ehrsson, H. H. How many arms make a pair? Perceptual illusion of having an additional limb. *Perception* **38**, 310–312 (2009).
2. Guterstam, A., Petkova, V. I. & Ehrsson, H. H. The illusion of owning a third arm. *PLOS ONE* **6**(2), e17208 (2011).
3. Kalckert, A. & Ehrsson, H. H. Moving a Rubber Hand that Feels Like Your Own: A Dissociation of Ownership and Agency. *Frontiers in human neuroscience* **6**, 40 (2012).
4. Armel, K. C. & Ramachandran, V. S. Projecting sensations to external objects: evidence from skin conductance response. *Proceedings. Biological sciences* **270**(1523), 1499–1506 (2003).
5. Ehrsson, H. H., Wiech, K., Weiskopf, N., Dolan, R. J. & Passingham, P. E. Threatening a rubber hand that you feel is yours elicits a cortical anxiety response. *Proceedings of the National Academy of Sciences of the United States of America* **104**(23), 9828–9833 (2007).
6. Ehrsson, H. H. et al. Upper limb amputees can be induced to experience a rubber hand as their own. *Brain: a journal of neurology* **131**(Pt 12), 3443–3452 (2008).
7. Petkova, V. I. & Ehrsson, H. H. When right feels left: referral of touch and ownership between the hands. *PLOS ONE* **4**(9), e6933 (2009).
8. Dawson, M. E., Schell, A. M. & Filion, D. L. The electrodermal system in *Handbook of Psychophysiology* (eds. Cacioppo, J. T., Tassinary, L. G. & Berntson, G.) Ch. 8, 200–223 (Cambridge University Press, 2006).
9. Lykken, D. T., Rose, R. J., Luther, B. & Maley, M. Correcting psychophysiological measures for individual differences in range. *Psychological Bulletin* **66**, 481–484 (1966).
10. Lenggenhager, B., Tadi, T., Metzinger, T. & Blanke, O. Video ergo sum: manipulating bodily self-consciousness. *Science* **317**, 1096–1099 (2007).
11. Ehrsson, H. H. The experimental induction of out-of-body experiences. *Science* **317**, 1048 (2007).
12. Noguchi, K., Gel, Y. R., Brunner, E., Konietschke, F. nparLD: An R Software Package for the Nonparametric Analysis of Longitudinal Data in Factorial Experiments. *Journal of Statistic Software* **50**(12), 1-23, (2012).
13. Bathke, A. C., Schabenberger, O., Tobias, R. D. & Madden, L. V. Greenhouse–Geisser Adjustment and the ANOVA-Type Statistic: Cousins or Twins? *The American Statistician* **63**(3), 239-246, (2009).
14. Brunner, E. & Puri, M. L. Nonparametric Methods in Factorial Designs. *Statistical Papers* **42**, 1-52, (2001).
15. Brunner, E., Domhof, S. & Langer, F. *Nonparametric Analysis of Longitudinal Data in Factorial Experiments.* (John Wiley & Sons, New York. 2002)
16. Lira, M. et al. The influence of skin colour on the experience of ownership in the rubber hand illusion. *Scientific Reports* **7**(1), 15745, (2017).
17. Costantini, M. & Haggard, P. The rubber hand illusion: Sensitivity and reference frame for body ownership. *Conscious. Cogn.* **16**, 229–240, (2007).
18. Tsakiris, M. My body in the brain: A neurocognitive model of body-ownership. *Neuropsychologia* **48**, 703–712 (2010).
19. Tsakiris, M. & Haggard, P. The rubber hand illusion revisited: visuotactile integration and self-attribution. *J. Exp. Psychol. Hum. Percept. Perform.* **31**, 80–91 (2005).
